# Supplementary material for: A versatile cohesion manipulation system reveals CENP-A dysfunction accelerates female reproductive age-related egg aneuploidy
Source: bioRxiv. 2025 Feb 27:2025.02.27.640570. Preprint. [Version 1] doi: 10.1101/2025.02.27.640570 (PMC11888391; doi:10.1101/2025.02.27.640570)
Supplement: 1 [file NIHPP2025.02.27.640570V1-supplement-1.pdf]

Fig. S1

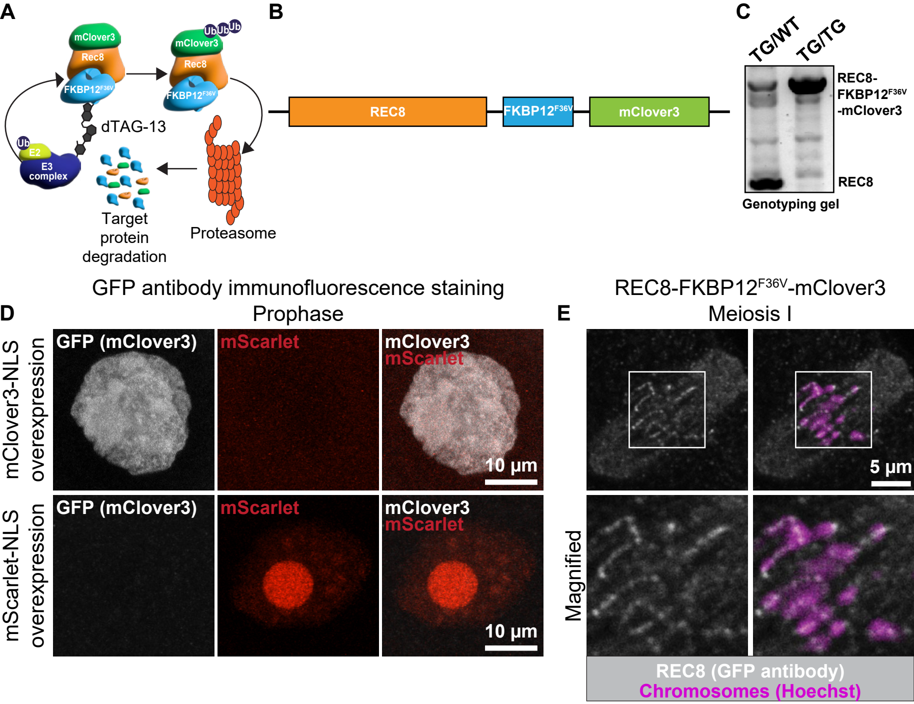
A
mClover3
Rec8
FKBP12F36V
Ub Ub Ub
dTAG-13
Ub
E2
E3 complex
Target protein degradation
Proteasome
B
REC8
FKBP12F36V
mClover3
C
TG/WT
TG/TG
REC8-FKBP12F36V-mClover3
REC8
Genotyping gel
GFP antibody immunofluorescence staining
Prophase
D
mClover3-NLS overexpression
mScarlet-NLS overexpression
GFP (mClover3)
mScarlet
mClover3
mScarlet
10 µm
E
REC8-FKBP12F36V-mClover3
Meiosis I
Magnified
5 µm
REC8 (GFP antibody)
Chromosomes (Hoechst)

bioRxiv preprint doi: https://doi.org/10.1101/2025.02.27.640570; this version posted February 27, 2025. The copyright holder for this preprint (which was not certified by peer review) is the author/funder, who has granted bioRxiv a license to display the preprint in perpetuity. It is made available under aCC-BY 4.0 International license.

Fig. S1. Generation of REC8-FKBP12^{F36V}-mClover3 mice as a versatile cohesion manipulation system.

(A) Graphical description of the principle of dTAG-13 proteolysis targeting chimera-mediated endogenous protein degradation.

(B) Graphical description of gene arrangements in REC8-FKBP12^{F36V}-mClover3 knockin mice. Boxes depicting genes are drawn to scale.

(C) A representative genotyping agarose gel analysis of REC8-FKBP12^{F36V}-mClover3 mouse genomic DNA using oligonucleotide pairs designed to distinguish between wild-type and knockin REC8 alleles.

(D) Representative maximum intensity projected immunofluorescence images showing that GFP antibodies specifically recognize mClover3 but not mScarlet in mClover3-NLS or mScarlet-NLS expressing prophase-arrested wild-type oocytes.

(E) Representative maximum intensity projected immunofluorescence images of REC8 (detected with GFP antibody) and chromosomes in metaphase I-stage oocytes isolated from REC8-FKBP12^{F36V}-mClover3 mice. Boxes represent regions of interest magnified in lower panels.

Fig. S2

A

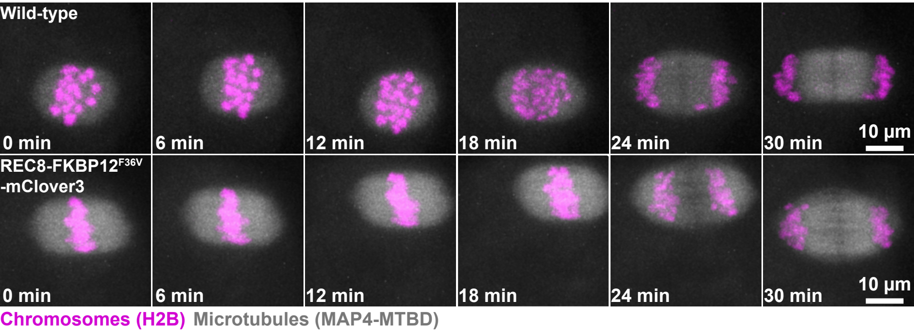

Wild-type
0 min
6 min
12 min
18 min
24 min
30 min
10 μm
REC8-FKBP12F36V
-mClover3
0 min
6 min
12 min
18 min
24 min
30 min
10 μm
Chromosomes (H2B)
Microtubules (MAP4-MTBD)

bioRxiv preprint doi: https://doi.org/10.1101/2025.02.27.640570; this version posted February 27, 2025. The copyright holder for this preprint (which was not certified by peer review) is the author/funder, who has granted bioRxiv a license to display the preprint in perpetuity. It is made available under aCC-BY 4.0 International license.

Fig. S2. CRISPR-Cas based C-terminal tagging of endogenous REC8 does not disrupt accurate oocyte chromosome alignment and segregation.

(A) Images from representative time lapse movies of chromosomes (marked with H2B-mScarlet) and meiotic spindles (marked with MAP4 microtubule-binding domain (mNeonGreen-MAP4-MTBD)) in wild-type and REC8-FKBP12^{F36V}-mClover3 oocytes.

Fig. S3

A

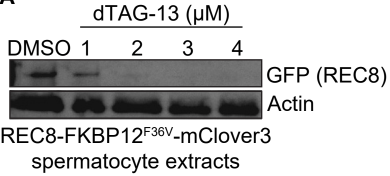
dTAG-13 (μM)
DMSO 1 2 3 4
GFP (REC8)
Actin
REC8-FKBP12^{F36V}-mClover3
spermatocyte extracts

bioRxiv preprint doi: https://doi.org/10.1101/2025.02.27.640570; this version posted February 27, 2025. The copyright holder for this preprint (which was not certified by peer review) is the author/funder, who has granted bioRxiv a license to display the preprint in perpetuity. It is made available under aCC-BY 4.0 International license.

Fig. S3. Validation of dTAG-13-mediated REC8 degradation in REC8-FKBP12^{F36V}-mClover3 spermatocytes.

(A) Western blotting analyses of REC8-FKBP12^{F36V}-mClover3 spermatocyte extracts treated with DMSO (control) or varying concentrations of dTAG-13. GFP antibody was used for detection of REC8 protein. Actin was used as loading control.

Fig. S4

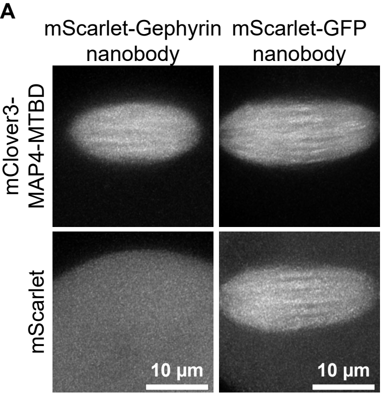
A
mScarlet-Gephyrin nanobody
mScarlet-GFP nanobody
mClover3-MAP4-MTBD
mScarlet
10 µm
10 µm

bioRxiv preprint doi: https://doi.org/10.1101/2025.02.27.640570; this version posted February 27, 2025. The copyright holder for this preprint (which was not certified by peer review) is the author/funder, who has granted bioRxiv a license to display the preprint in perpetuity. It is made available under aCC-BY 4.0 International license.

Fig. S4. GFP-nanobodies specifically recognize mClover3 protein.

A) High-resolution images of live, metaphase II-arrested wild-type mouse eggs co-expressing mClover3-MAP4-MTBD and mScarlet-tagged Gephyrin or GFP nanobodies.

Fig. S5

A

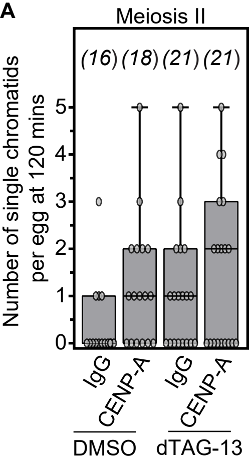
Meiosis II
(16) (18) (21) (21)
Number of single chromatids per egg at 120 mins
0
1
2
3
4
5
IgG
CENP-A
IgG
CENP-A
DMSO
dTAG-13

bioRxiv preprint doi: https://doi.org/10.1101/2025.02.27.640570; this version posted February 27, 2025. The copyright holder for this preprint (which was not certified by peer review) is the author/funder, who has granted bioRxiv a license to display the preprint in perpetuity. It is made available under aCC-BY 4.0 International license.

Fig. S5. CENP-A TRIM-away increases the number of PSCS in dTAG-13-mediated cohesion degradation.

(B) Quantification of the number of single chromatids per egg at 120 mins of live imaging in DMSO-treated and IgG TRIM-Away, DMSO-treated and CENP-A TRIM-Away, dTAG-13-treated and IgG TRIM-Away or dTAG-13-treated and CENP-A TRIM-Away eggs from REC8-FKBP12^{F36V}-mClover3 mice.

bioRxiv preprint doi: https://doi.org/10.1101/2025.02.27.640570; this version posted February 27, 2025. The copyright holder for this preprint (which was not certified by peer review) is the author/funder, who has granted bioRxiv a license to display the preprint in perpetuity. It is made available under aCC-BY 4.0 International license.

Table S1. Oligonucleotide sequences used in this study.

Primer Name	Primer Sequence
BM729	TCGATGCCCTTCAGCTCGAT
BM730	CCACCCTCGTGACCACCT
BM732	ggctgctacagcacactcta
BM733	CCACCCCGGTGAACAGCT
BM734	gttatcccagctgacacccc
BM735	aaaggtggcctgtcttctgc
KS109	agcgctaccggtctcagatcATGCCAGAGCCAGCGAAG
KS110	tcccgggccgtcgactgcagaattCTTAGCGCTGGTGTACTTGG
KS156	gctgtacaagcttaaggagttcgtgaccgccgc
KS157	tgctcaccatggtggcgaccggtagcgc
KS158	ctagcgctaccggtcgccaccATGGTGAGCAAGGGCGAG
KS159	cggcggcggtcacgaactccttaagCTTGTACAGCTCGTCCATGC
KS111	ggtcgccaccatggtgagcaagggcgag
KS112	actccttaagcttgtacagctcgtccatgc
BM343	ggtctcagatctcgagctcaATGGATCAAGTCCAACTGGTG
BM344	tcagatctaaccatctgcagGCTGGAGACGGTGACCTG
JL7	ggtctcagatctcgagctcaATGGCCGAGGTGCAGCTG
JL8	tcagatctaaccatctgcagTGCGCCATGGTGATGGTG
BM715	CTTGTACAGCTCGTCCATG
BM811	tggacgagctgtacaagatcATGGCAGAACAAGGGGCC
BM812	aattcgaagcttgagctcgaCTAGATCACCTCGGCCGTTTG
BM813	GTGCAGGCAGAAGATGAAG
JL9	AGGAATTCGATATCAAGCTTC
JL10	AGCTCGAGATCTGAGACC
JL3	ccggtctcagatctcgagctATGGCCGAGGTGCAGCTG
JL4	aagcttgatatcgaattcctTGCGCCATGGTGATGGTG
BM432	ggtctcagatctcgagctcaATGGTGAGCAAGGGCGAG
BM433	ccgtcgactgcagaattcgaTTACACCTTCCGCTTTTTCTTAGG
FG39	CTGCAGGGGATGGTGAGC
BM494	TGAGCTCGAGATCTGAGAC
FG40	CACCTTCCGCTTTTTCTTAGGC

bioRxiv preprint doi: https://doi.org/10.1101/2025.02.27.640570; this version posted February 27, 2025. The copyright holder for this preprint (which was not certified by peer review) is the author/funder, who has granted bioRxiv a license to display the preprint in perpetuity. It is made available under aCC-BY 4.0 International license.

Movie S1. Navigation through 1 μm apart confocal sections of REC8 (GFP antibody, grey) and homologous chromosomes (magenta) in a metaphase I-stage mouse oocyte.

Movie S2. High-resolution time lapse movie showing the selective removal of REC8 from chromosome arms and its retention at centromeric regions during anaphase I in a REC8-FKBP12^{F36V}-mClover3 mouse oocyte.

Movie S3. Time lapse movie of chromosome alignment and segregation during meiosis I in a wild-type mouse oocyte. Microtubules (grey) are labeled with mNeonGreen-MAP4-MTBD and chromosomes (magenta) are labeled with H2B-mScarlet.

Movie S4. Time lapse movie of chromosome alignment and segregation during meiosis I in a REC8-FKBP12^{F36V}-mClover3 mouse oocyte. Microtubules (grey) are labeled with mNeonGreen-MAP4-MTBD and chromosomes (magenta) are labeled with H2B-mScarlet.

Movie S5. Time lapse movie of chromatids (H2B-mScarlet, grey) in a DMSO-treated (dTAG-13 control), metaphase II-arrested REC8-FKBP12^{F36V}-mClover3 egg.

Movie S6. Time lapse movie of chromatids (H2B-mScarlet, grey) in a dTAG-13-treated, metaphase II-arrested REC8-FKBP12^{F36V}-mClover3 egg.

Movie S7. Time lapse movie of chromatids (H2B-mScarlet, grey) in a Gephyrin TRIM-Away, metaphase II-arrested REC8-FKBP12^{F36V}-mClover3 egg.

Movie S8. Time lapse movie of chromatids (H2B-mScarlet, grey) in a GFP TRIM-Away, metaphase II-arrested REC8-FKBP12^{F36V}-mClover3 egg.

Movie S9. Time lapse movie of chromatids (H2B-mScarlet, grey) in a DMSO-treated (CytoD control), metaphase II-arrested REC8-FKBP12^{F36V}-mClover3 egg.

Movie S10. Time lapse movie of chromatids (H2B-mScarlet, grey) in a CytoD-treated, metaphase II-arrested REC8-FKBP12^{F36V}-mClover3 egg.

bioRxiv preprint doi: https://doi.org/10.1101/2025.02.27.640570; this version posted February 27, 2025. The copyright holder for this preprint (which was not certified by peer review) is the author/funder, who has granted bioRxiv a license to display the preprint in perpetuity. It is made available under aCC-BY 4.0 International license.

Movie S11. Time lapse movie of chromatids (H2B-mScarlet, grey) in a dTAG-13-treated, metaphase II-arrested REC8-FKBP12^{F36V}-mClover3 egg.

Movie S12. Time lapse movie of chromatids (H2B-mScarlet, grey) in a CytoD- and dTAG-13-treated, metaphase II-arrested REC8-FKBP12^{F36V}-mClover3 egg.

Movie S13. Time lapse movie of chromatids (H2B-mScarlet, grey) in a DMSO-treated and IgG TRIM-Away metaphase II-arrested REC8-FKBP12^{F36V}-mClover3 egg.

Movie S14. Time lapse movie of chromatids (H2B-mScarlet, grey) in a DMSO-treated and CENP-A TRIM-Away metaphase II-arrested REC8-FKBP12^{F36V}-mClover3 egg.

Movie S15. Time lapse movie of chromatids (H2B-mScarlet, grey) in a dTAG-13-treated and IgG TRIM-Away metaphase II-arrested REC8-FKBP12^{F36V}-mClover3 egg.

Movie S16. Time lapse movie of chromatids (H2B-mScarlet, grey) in a dTAG-13-treated and CENP-A TRIM-Away metaphase II-arrested REC8-FKBP12^{F36V}-mClover3 egg.
